# Assessing Hepatitis C Burden and Treatment Effectiveness through the British Columbia Hepatitis Testers Cohort (BC-HTC): Design and Characteristics of Linked and Unlinked Participants

**DOI:** 10.1371/journal.pone.0150176

**Published:** 2016-03-08

**Authors:** Naveed Zafar Janjua, Margot Kuo, Mei Chong, Amanda Yu, Maria Alvarez, Darrel Cook, Rosemary Armour, Ciaran Aiken, Karen Li, Seyed Ali Mussavi Rizi, Ryan Woods, David Godfrey, Jason Wong, Mark Gilbert, Mark W. Tyndall, Mel Krajden

**Affiliations:** 1 British Columbia Centre for Disease Control, Vancouver, BC, Canada; 2 School of Population and Public Health, University of British Columbia, Vancouver, BC, Canada; 3 British Columbia Ministry of Health, Victoria, BC, Canada; 4 Performance Measurement & Reporting, Provincial Health Services Authority, Vancouver, BC, Canada; 5 BC Cancer Agency, Vancouver, BC, Canada; 6 Ontario HIV Treatment Network, Toronto, ON, Canada; 7 Department of Pathology and Laboratory Medicine, University of British Columbia, Vancouver, BC, Canada; University of Cincinnati College of Medicine, UNITED STATES

## Abstract

**Background:**

The British Columbia (BC) Hepatitis Testers Cohort (BC-HTC) was established to assess and monitor hepatitis C (HCV) epidemiology, cost of illness and treatment effectiveness in BC, Canada. In this paper, we describe the cohort construction, data linkage process, linkage yields, and comparison of the characteristics of linked and unlinked individuals.

**Methods:**

The BC-HTC includes all individuals tested for HCV and/or HIV or reported as a case of HCV, hepatitis B (HBV), HIV or active tuberculosis (TB) in BC linked with the provincial health insurance client roster, medical visits, hospitalizations, drug prescriptions, the cancer registry and mortality data using unique personal health numbers. The cohort includes data since inception (1990/1992) of each database until 2012/2013 with plans for annual updates. We computed linkage rates by year and compared the characteristics of linked and unlinked individuals.

**Results:**

Of 2,656,323 unique individuals available in the laboratory and surveillance data, 1,427,917(54%) were included in the final linked cohort, including about 1.15 million tested for HCV and about 1.02 million tested for HIV. The linkage rate was 86% for HCV tests, 89% for HCV cases, 95% for active TB cases, 48% for HIV tests and 36% for HIV cases. Linkage rates increased from 40% for HCV negatives and 70% for HCV positives in 1992 to ~90% after 2005. Linkage rates were lower for males, younger age at testing, and those with unknown residence location. Linkage rates for HCV testers co-infected with HIV, HBV or TB were very high (90–100%).

**Conclusion:**

Linkage rates increased over time related to improvements in completeness of identifiers in laboratory, surveillance, and registry databases. Linkage rates were higher for HCV than HIV testers, those testing positive, older individuals, and females. Data from the cohort provide essential information to support the development of prevention, care and treatment initiatives for those infected with HCV.

## Introduction

Hepatitis C virus (HCV) is a major global public health problem with ~184 million people infected worldwide. In Canada, between 230,000–450,000 (0.66% -1.3%) people are infected with HCV [1]. Most were infected decades ago and are now increasingly presenting with HCV-related sequelae (e.g., cirrhosis, end-stage liver disease and liver cancer).

Although potentially curative HCV treatments have been available since 2000, uptake and effectiveness has been low. Newer, short-course, well-tolerated direct acting antiviral therapies are highly effective (95%) in curing HCV but are very expensive [2–6]. Accurate and up-to-date knowledge about the current and future burden of disease, co-infections with HIV and HBV, health disparities, and cost of HCV-related illness relative to the cost and effectiveness of treatment are needed to inform public funding decisions for the newer antiviral drug therapies; the need for population level screening; and to prioritize resources for engaging infected individuals into care and treatment. Furthermore, there is a need for systems to monitor effectiveness and the overall impact of newly approved treatments on long-term outcomes.

The British Columbia (BC) Ministry of Health (MoH) approved a comprehensive linkage of public health surveillance and laboratory data with administrative healthcare data to create the BC Hepatitis Testers Cohort (BC-HTC). The *purpose of the cohort is to* assess and monitor HCV disease burden; HCV/HIV/HBV/TB co-infections; syndemics; disparities in testing and care; health care utilization; treatment uptake and completion; effectiveness of interferon-based and newer non-interferon-based treatments; cost of HCV related illness and impact of treatment on illness related costs and outcomes.

This paper describes the cohort construction, data linkage process and yield, and comparison of the characteristics of linked and unlinked individuals. The linkage of multiple public health surveillance and laboratory databases included in the cohort is based on both deterministic and probabilistic linkage algorithms. Individuals in the cohort were deterministically linked with administrative health care databases using a unique personal health number (PHN). At each stage, some records with missing identifiers did not get linked and, hence, did not become part of the final dataset. Persons at the highest risk of HCV and/or HIV may be more likely to have missing identifying information needed for linkage, which may lead to selection bias and may affect the generalizability of results from the analysis of the cohort. Thus, it is important to understand the differences between those who met the cohort inclusion criteria and those who finally remained in the linked dataset, in order to understand biases affecting future analyses. This paper describes the linkage process and characteristics of linked and un-linked individuals, providing essential background for interpreting findings from this cohort.

## Methods

### BC Hepatitis Testers Cohort

**Fig 1:**
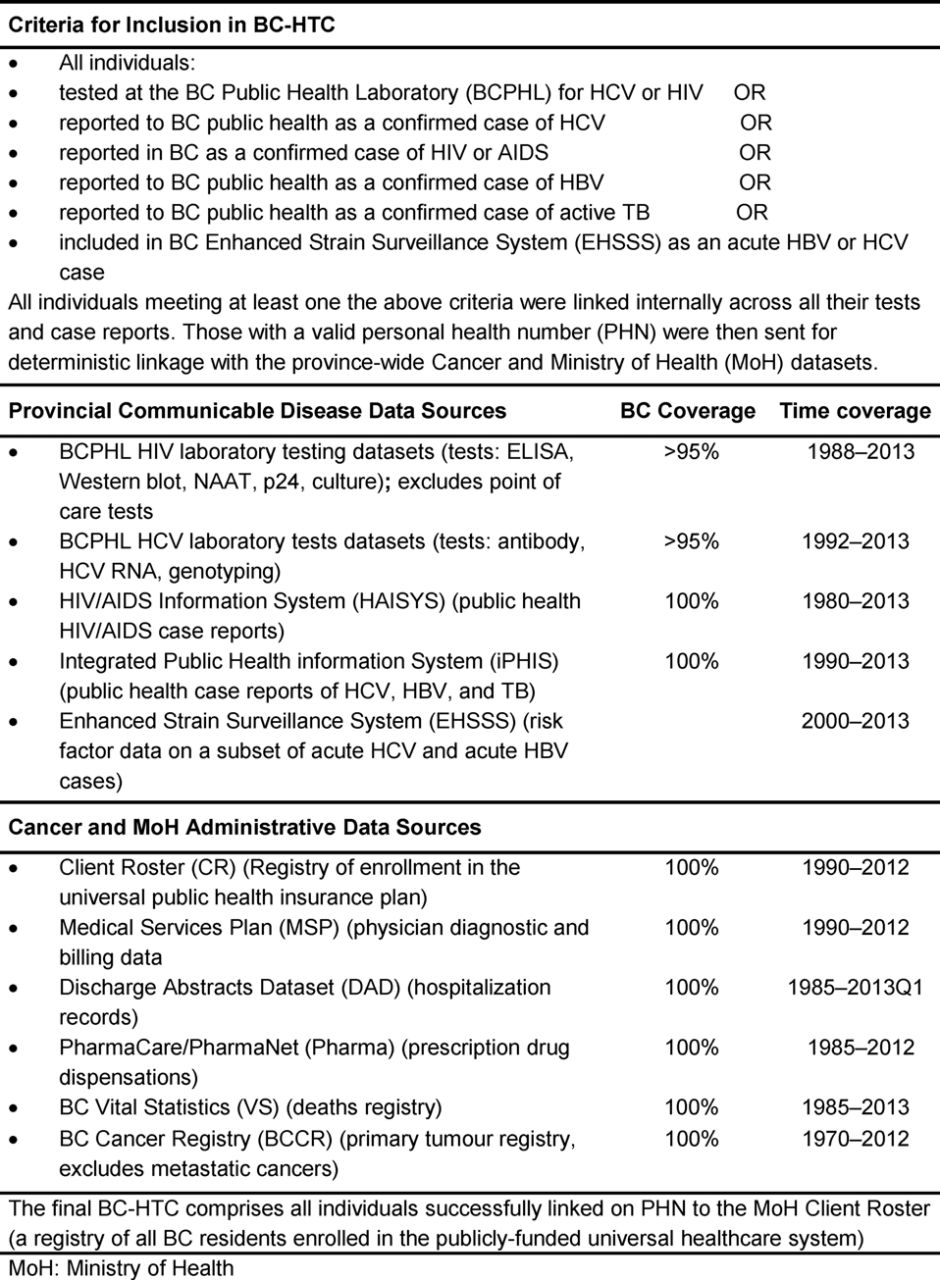
Criteria and Data Sources for the BC Hepatitis Testers Cohort (BC-HTC).

The BC Hepatitis Testers Cohort (BC-HTC) includes all individuals who have been tested for HCV or HIV at the BC Public Health Laboratory (BCPHL) or who have been reported to public health as a case of HCV, hepatitis B (HBV), HIV or active tuberculosis (TB) in BC, Canada. The laboratory/public health surveillance data are linked with the BC MoH Client Roster, medical visits, hospitalizations, prescription drug data, cancer registry and mortality (**Fig 1**). The cohort includes a longitudinal linkage of records from inception (1990) of each dataset to 2012/13, with plans for annual updates thereafter. The resulting linked cohort includes data on more than 1.5 million individuals, about a third of BC’s 4.6 million population.

### Data access and ethics approval

Data linkages were approved by the data stewards of the BC Centre for Disease Control (BCCDC), the BCPHL, the BC MoH, the BC Vital Statistics Agency and the BC Cancer Registry. Approval for data linkage with the MoH datasets was granted under the BCCDC public health mandate to conduct surveillance and program evaluation. The study was reviewed and approved by the University of British Columbia Behavioral Research Ethics Board (No: H14-01649).

### Case definitions and identification

As an HCV positive case was identified by multiple laboratory tests and/or public health case reports, algorithms were created to define HCV cases and assign a single diagnosis date. An individual testing anti-HCV positive, HCV RNA positive, genotype positive or who was reported as an HCV case in the Integrated Public Health Information System (iPHIS) was considered a HCV case in this cohort [7]. An individual included in the HIV/AIDS Information System (HAISYS), or who had positive HIV lab test results was considered a case based on provincial HIV laboratory test interpretation guidelines [8]. To capture additional HIV cases who may have been tested without nominal information and hence may not have been captured from HAISYS or laboratory data, a previously validated algorithm requiring at least three Medical Services Plan (MSP) visits with an HIV diagnosis code or a hospital admission with an HIV diagnosis code was also considered an HIV case (**Table 1 and S2 Fig**) [9]. HBV and active TB diagnosis was based on provincial guidelines [10, 11].

**Table 1:** Case definitions for HCV, HBV, HIV and active TB.

Infection	Case definition
** HCV Case**	a confirmed anti-HCV positive test OR
	a confirmed HCV RNA positive test OR
	an HCV genotype result OR
	a confirmed public health HCV case report in iPHIS [7]
**HIV Case**	Any one of the following was considered an HIV case:
	1. Laboratory confirmation based on provincial HIV test interpretation guidelines [8]:
	HIV antibody by screening test (i.e., ELISA) followed by positive confirmatory test (i.e., Western Blot or Nucleic Acid Amplification Test), OR
	detection of HIV nucleic acid (RNA or DNA) OR
	detection of p24 antigen with confirmation by neutralization assay, OR
	isolation of HIV in culture.
	2. a confirmed HIV case report in HAISYS
	3. three physician claims or one hospital admission with diagnosis of HIV using ICD 9/10 codes: MSP: ICD-9 code starting with 042, 043, 044, or codes V08, 795.71, 795.8 or 07953; DAD: ICD9–CM codes starting with 042, 043, 044, or codes V08, 795.71, 795.8 or 07953; ICD-10-CA codes: B20–B24, F024, R75, Z21, O987, B9735.
** HBV Case**	a confirmed public health HBV case report recorded in iPHIS defined on the basis of provincial guidelines [10].
** Active TB Case**	a confirmed public health active TB case report recorded in iPHIS [11].

iPHIS: Integrated Public Health Information System; HAISYS: HIV/AIDS Information System; MSP: Medical Services Plan; DAD: Discharge Abstract Database.

### Data sources

The following data sources are included in the cohort (**Fig 1**):

#### Laboratory and public health surveillance databases

BCPHL Laboratory Information System: Greater than 95% of all anti-HCV tests and all HCV RNA testing and genotyping conducted in BC are performed at the BCPHL. This dataset includes all laboratory testing records of persons tested for HCV since 1992 and HIV tests performed since 1988. HCV data include antibody screening and confirmatory tests, genotype results and HCV RNA data (both qualitative and quantitative viral load testing).

iPHIS stores information on all cases of HCV, HBV and TB reported to public health in BC, including a small number of cases not tested at BCPHL.

The BCCDC Enhanced Hepatitis Strain Surveillance System (EHSSS) includes detailed risk factor data on a subset of acute HBV and acute HCV cases identified between 2000–2013.

HAISYS contains data on new diagnoses of HIV/AIDS reported to public health together with enhanced risk factor data. Of note, in BC, there is the option for non-nominal reporting of HIV in which a positive HIV test result is reported to public health with the client’s initials only and without an address. About 1% of unique clients in HAISYS have chosen non-nominal reporting at least once.

#### Administrative healthcare databases

The Client Roster contains information on all BC residents enrolled in the publicly funded health care plan. Each person is assigned a unique PHN which serves as a unique identifier in all healthcare databases. In addition to demographic information, extracted data include the number of days registered with the plan and the residential six digit postal code for every year of registration.

MSP: MSP is BC’s universal health insurance plan that reimburses medically required services provided to individuals by fee-for-service practitioners. MSP data includes all encounters and associated International Classification of Disease (ICD) 9 diagnostic codes. MSP records on linked cohort members are available since January 1, 1990 [12].

Discharge Abstract Database (DAD): DAD compiles data on all hospitalizations in BC and records on linked cohort members are available from April 1, 1985 [13].

PharmaCare/PharmaNet: PharmaNet is the province-wide network managed by BC MoH and the College of Pharmacists of BC that links pharmacies to a central data system in which prescription drugs dispensed in BC are recorded. PharmaCare is the BC public prescription drug insurance plan that assists BC residents in paying for eligible prescription drugs. PharmaCare data ranges from January 1, 1985 to December 31, 1995. PharmaNet data starts from January 1, 1996 and includes the types of data previously recorded in PharmaCare [14].

Vital Statistics mortality data: BC Vital Statistics Agency captures information on all deaths that occur in BC. Out of province deaths of BC residents are not included. Death records on linked cohort members include the date of death, underlying cause(s) of death, and antecedent cause(s) of death since January 1, 1985 [15].

The BC Cancer Registry (BCCR) compiles data on cancer incidence and mortality including demographics and detailed information on cancer diagnoses (e.g. cancer site, histology, date of diagnosis) since 1970 [16].

### Linkage of public health surveillance, laboratory and administrative datasets

The BC-HTC was created within the Public Health Reporting Data Warehouse (PHRDW) at BCCDC. PHRDW is a public health data warehouse for linking BCPHL and BCCDC datasets to provide role-based access to data and summary reports. The PHRDW data linkage algorithm was used for patient-matching to identify unique individuals within and across datasets, including HCV and HIV laboratory data, HIV cases from HAISYS, and HCV, HBV and TB cases from iPHIS. There are three levels of matching within PHRDW using different levels of linkage certainty. Level 1 corresponds to perfect (matches on: PHN, First Name + Last Name + Date of Birth+ Gender), level 2 to strong (PHN, First Name + Last Name + Date of Birth checked), and level 3 to a moderate match (PHN + Date of Birth checked). For level 3 match, date of birth check means, birth year for all records in a matched set is same, but difference in month or day is allowed. When PHN is not available for a record, matching is based on First Name + Last Name + Date of Birth+ Gender. For BC-HTC generation, level 3 matching was used. At this stage, a unique BCCDC ID was generated and assigned to each linkable individual in the cohort. The validity of this approach was assessed when the warehouse was developed [17]. A cohort linkage file was generated from the linked dataset. Records with missing or invalid PHNs and missing demographic information were deemed unlinkable for further linkage and were not sent for matching with the Client Roster at MoH.

The cohort linkage file including the BCCDC ID, PHN and demographic information was sent to the MoH. At the MoH, records were matched with the Client Roster using PHN, date of birth and gender. A MoH study ID was then appended to all matched records and a crosswalk file linking the BCCDC ID and the MOH study ID was created. Each MoH data source and BCCR then extracted content data on the matched individuals, leaving only the MoH study ID as a unique ID. The crosswalk file was sent to the BCCDC to be used to append the MoH study ID to BCCDC content data (HCV, HIV, HBV, and TB disease status). At this stage, BCCDC ID and other identifiers were removed from the BCCDC content data. The MoH and BCCR data with only MoH study ID was sent to BCCDC. The only unique identifier shared across all datasets is the MoH study ID; linked data in the BC-HTC does not include any identifiers. The MoH study ID will be used for further linkages and annual updates (**S1 Fig**).

### Follow-up

The current BC-HTC dataset provides testing, co-infection, comorbidity and outcome data for about 20 years (1992-2012/13) on individuals tested in 1992 and a shorter follow-up period for those tested thereafter. Annual updates of the linked dataset through the same linkage process will allow inclusion of new testers and follow-up of individuals already included in the cohort.

### Data management and storage

The linkage produced a large dataset with various subsets exceeding 100 gigabytes. We selected an SQL server-based relational database where the various datasets are stored in separate tables and joined together through a unique key (Study ID). This system allows creation of data views which present a subset of data for analysis, thus enhancing analysis speed and efficiency. It also facilitates construction of a robust security system where analysts’ access to the dataset is tailored to the needs. The dataset is accessed for analysis by connecting analysis software such as SAS or R through an Open Database Connectivity (ODBC) connection.

## Results

### Linkage yield

**Fig 2:**
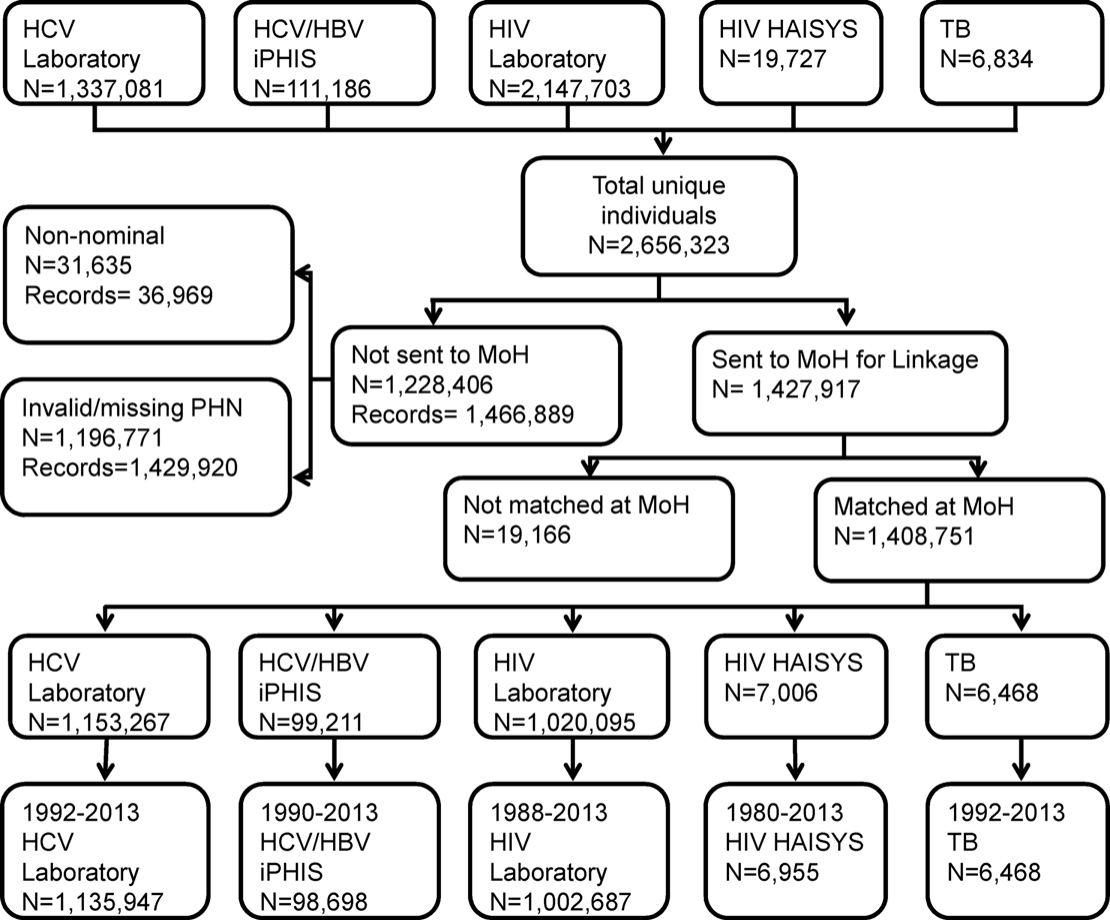
Flow diagram showing overall linkage yield.

There were 2,656,323 individuals available from all data sources contributing to the BC-HTC. Of these, 1,427,917 (54%) were sent to the MoH for linkage with the Client Roster. The remaining 1,228,204 individuals either did not have valid PHN or tests were submitted non-nominally for HIV testing. Of those sent to the MoH for linkage a very small percentage (n = 19,166; 1.3%) did not match with the Client Roster (**Fig 2**) due to mismatch between PHN recorded in the laboratory or surveillance data and the Client Roster. In the linked cohort about 1.15 million individuals were tested for HCV and 1.02 million for HIV.

The percentages of individuals within each dataset with valid data available for linkage and sent to the MoH for linkage are presented in **Table 2**. The table shows that the percentage sent for linkage was >85% for HCV testing and case data while it was ~50% for HIV testing and 35% for HIV case data. The linkage yield was 86% for HCV test data, 89% for hepatitis case data, 95% for active TB cases, 48% for HIV test data and 36% for HIV case data **(Table 2).** Major reasons for lower linkage of HIV data relate to lack of PHN and other identifiers needed for linkage during the earlier years of the HIV epidemic. PHNs were not recorded regularly in the laboratory information system prior to 2006. Availability of PHN and other demographic data improved after HIV became reportable in BC in 2003.

**Table 2:** Number of total and linked records and unique individuals in various data sources constituting the BC-HTC, Canada.

Infection	Data set	Total	Sent to MoH ^a^	Linked	% linked(sent)^c^	% linked (all)^b^
	**Unique Individuals**					
HCV/HBV	HCV Laboratory	1,337,081	1,161,537	1,153,267	99.3%	86.3%
	HCV/HBV cases iPHIS	111,186	100,105	99,211	99.1%	89.2%
	EHSSS data	1,412	1,389	1,386	99.8%	98.2%
	EHSSS Risk factors HBV	324	320	320	100.0%	98.8%
	EHSSS Risk factors HCV	462	455	455	100.0%	98.5%
HIV	HIV Laboratory	2,147,703	1,024,655	1,020,095	99.6%	47.5%
	HIV cases HAISYS	19,727	7,017	7,006	99.8%	35.5%
TB	Active TB cases	6,834	6,490	6,468	99.7%	94.6%
** **	**ALL Unique Patients**	**2,656,323**	**1,427,917**	1,408,751	**98.7%**	**53.0%**
	**Unique Records** ^d^					
HCV/ HBV	HCV Laboratory	2,338,308	2,125,778	2,115,566	99.5%	90.5%
	HCV/HBV cases iPHIS	121,285	109,783	108,711	99.0%	89.6%
	EHSSS data	1,415	1,392	1,389	99.8%	98.2%
	EHSSS Risk factors HBV	325	321	321	100.0%	98.8%
	EHSSS Risk factors HCV	462	455	455	100.0%	98.5%
HIV	HIV Laboratory	3,517,629	2,222,380	2,215,689	99.7%	63.0%
	HIV cases HAISYS	19,988	7,206	7,195	99.8%	36.0%
TB	Active TB cases	6,955	6,610	6,587	99.7%	94.7%
** **	**ALL Unique Records**	**4,556,811**	**3,089,922**	3,075,683	**99.5%**	**67.5%**

EHSSS: Enhanced Hepatitis Strain Surveillance System; iPHIS: Integrated Public Health Information System; HAISYS: HIV/AIDS Information System; MoH: Ministry of Health

^a^ Sent to MoH for linkage with the Client Roster

^b^ Sent to MoH for linkage with the Client Roster /Total

^c^ Linked/total

^d^ Over a period of time there are individuals testing multiple times and thus have multiple records per person.

In defining and identifying HIV positive cases, 6955 cases were identified from HAISYS, 7427 from lab data, and 9983 were identified using MSP and DAD for a total of 11025 HIV cases (**S2 Fig).** Most of the cases (5795, 53%) were present in all 3 sources, 932 (8%) were present in lab data and HAISYS; 666(6%) in lab data and MSP/DAD; 197 (2%) in HAISYS and MSP/DAD while 3325 (30%) were only recorded in MSP/DAD.

### Loss/completion rate by year

**Fig 3:**
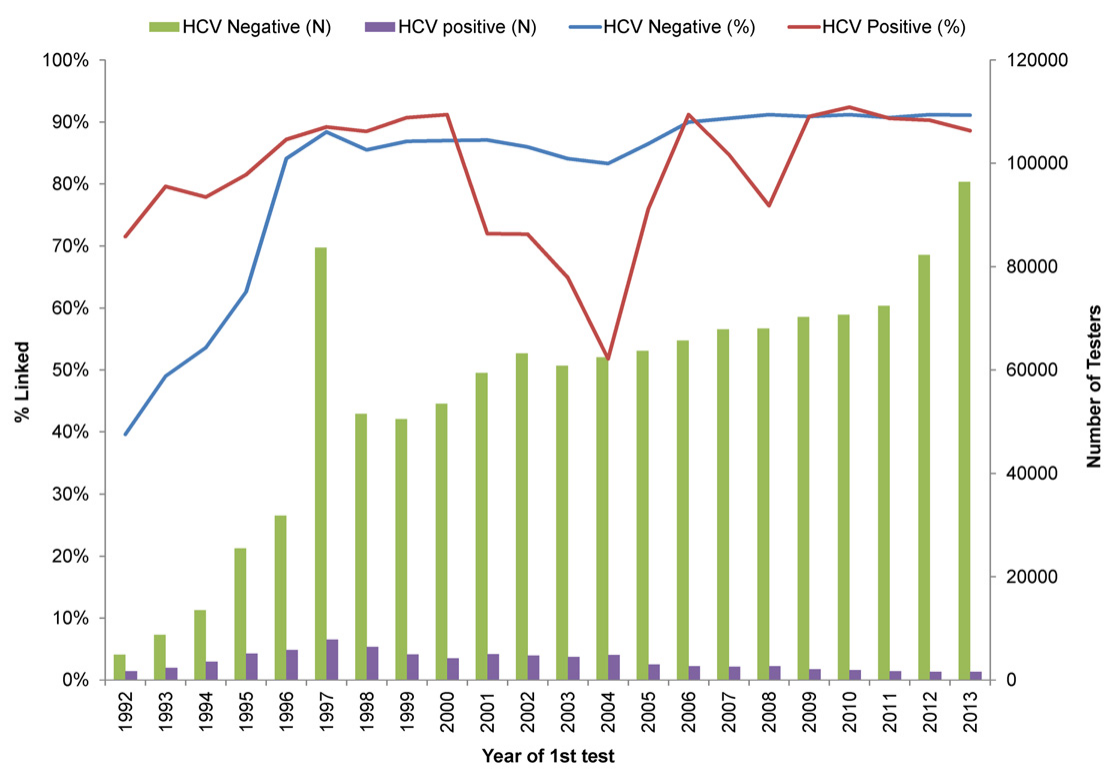
HCV laboratory data linkage rate by year 1992–2013.

**Fig 4:**
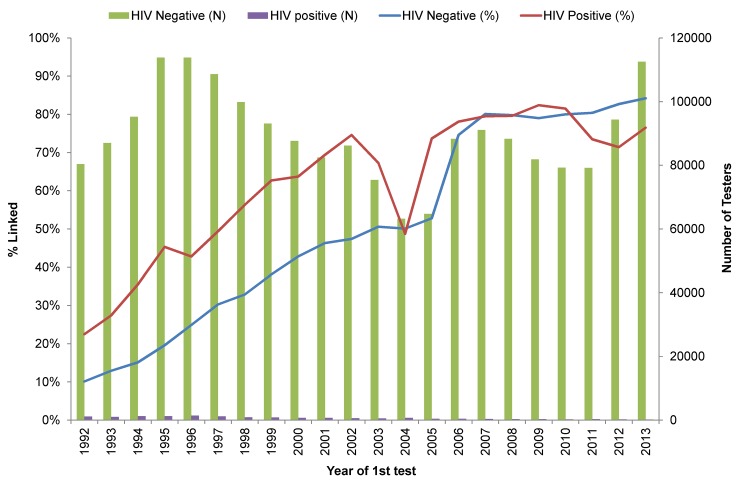
HIV laboratory data linkage rate by year 1992–2013.

In the earlier years, linkage rates were low for both HCV and HIV (**Fig 3 and Fig 4**). For HCV negatives, the linkage rate increased from 40% in 1992 to 84% in 1996 and stayed in this range until 2005 after which the rate was ~90%. For HCV positives, the linkage rate gradually increased from 72% in 1992 to 90% in 1999 and remained at ~90% with the exception of 2000–2005 where it dropped to 70% (**Fig 3**). The HIV linkage rate followed a similar trend except that the linkage rate for HIV negatives was 10% in 1992, gradually increased to 50% in 2005 and thereafter stayed at ~80%. For HIV positives, the linkage rate was more variable starting with 23% in 1992, increasing to 64% in 2000 and then ranged between 69–83% until 2013, with the exception of 2004 when it dropped to 49% (**Fig 4**). When HCV cases were defined based on anti- HCV testing only, the linkage rate for positives dropped only in 2004 rather than from 2000–2005 as occurred for the integrated anti-HCV and RNA test results (**S3 Fig**). The drop in linkage rates for both anti-HCV positive and HIV positive groups in 2004 and for HCV in 2000–2004 relates to lack of identifiers accompanying specimens submitted as part of research projects.

### Characteristics of cohort and comparison with those not linked

#### Hepatitis C Testers

There was a difference in the distribution of linked and unlinked HCV cases by year of first test (**Table 3**). The highest proportion of linked HCV cases (34%) was tested during 1996–1999 while the highest proportion of unlinked cases (50.5%) was tested from 2000–2005. Among HCV negative testers, the highest proportions of linked and not linked testers were from recent years (2006–2013: 50.5% vs. 34.2%) and the lowest were from the earliest years (1992–1995: 2.8% vs. 14.7%). Most of the RNA tests (70%) in the linked group were performed in 2006–2013 while in the unlinked groups the majority (65%) were performed in 2000–2005 (**Table 3**).

**Table 3:** Comparison of linked and unlinked HCV testers.

	HCV Positive				HCV Negative			
	Linked		Unlinked	Overall	Linked		Unlinked	Overall
	N(%)	% linked	N(%)	N(%)	N(%)	% linked	N(%)	N(%)
**N**	67726	79	17971	85697	1068221	87.1	158325	1226546
**N**^**a**^	65834	81	15377	81211				
**Year of 1st HCV test** ^a^								
1992–1995	10104(15.3)	78.8	2723(17.7)	12827(15.8)	29443(2.8)	55.9	23252(14.7)	52695(4.3)
1996–1999	22303(33.9)	88.8	2802(18.2)	25105(30.9)	188643(17.7)	86.7	28821(18.2)	217464(17.7)
2000–2005	18631(28.3)	70.6	7762(50.5)	26393(32.5)	310750(29.1)	85.6	52156(32.9)	362906(29.6)
2006–2013	14796(22.5)	87.6	2090(13.6)	16886(20.8)	539385(50.5)	90.9	54096(34.2)	593481(48.4)
**Year of 1**^**st**^ **+/ last–ve HCV test** ^a^								
1992–1995	9232(13.6)	72.3	3541(19.8)	12773(14.9)	11859(1.1)	35.1	21923(13.8)	33782(2.8)
1996–1999	20877(30.9)	83.3	4200(23.5)	25077(29.3)	125217(11.7)	82.2	27030(17.1)	152247(12.4)
2000–2005	19275(28.5)	70.9	7923(44.3)	27198(31.8)	240353(22.5)	82.3	51625(32.6)	291978(23.8)
2006–2013	18273(27)	89.2	2218(12.4)	20491(24)	690792(64.7)	92.3	57747(36.5)	748539(61)
**RNA PCR performed** ^a^								
1992–1995	666(0.6)	84.8	119(1.3)	785(0.6)	80(0.6)	48.8	84(1.1)	164(0.8)
1996–1999	2538(2.1)	97.2	74(0.8)	2612(2)	773(5.9)	76.1	243(3.2)	1016(4.9)
2000–2005	33131(27.6)	84.3	6169(65.3)	39300(30.4)	4398(33.6)	39.7	6667(86.8)	11065(53.3)
2006–2012	83531(69.7)	96.4	3080(32.6)	86611(67)	7822(59.8)	91.9	686(8.9)	8508(41)
**Genotype performed** ^a^								
1992–1995	0		0	0				
1996–1999	21(0.1)	100	0	21(0.1)				
2000–2005	13120(39)	78.8	3539(86.2)	16659(44.2)				
2006–2013	20482(60.9)	97.3	565(13.8)	21047(55.8)				
**Last genotype on record** ^a^								
GT1	18600(61.4)	87.7	2616(65.9)	21216(61.9)				
GT2	3813(12.6)	89.6	444(11.2)	4257(12.4)				
GT3	7368(24.3)	90.5	774(19.5)	8142(23.8)				
GT4-6	328(1.1)	71.6	130(3.3)	458(1.3)				
Mixed	186(0.6)	96.9	6(0.2)	192(0.6)				
**Birth year**								
1900–1945	6365(9.4)	81.4	1450(8.1)	7815(9.1)	154199(14.4)	87.9	21159(13.4)	175358(14.3)
1945–1964	41298(61)	80.1	10284(57.2)	51582(60.2)	327477(30.7)	88.3	43418(27.4)	370895(30.2)
1965–1974	12977(19.2)	79.5	3345(18.6)	16322(19)	212498(19.9)	87.9	29207(18.4)	241705(19.7)
After 1975	7085(10.5)	84.2	1327(7.4)	8412(9.8)	374018(35)	86.6	57987(36.6)	432005(35.2)
Unknown	1(0)	0.1	1565(8.7)	1566(1.8)	29(0)	0.4	6554(4.1)	6583(0.5)
**Age at first test** ^a^								
<25 yrs	5860(8.9)	86.5	911(6)	6771(8.3)	217442(20.4)	84.8	38951(25.7)	256393(21)
25–34 yrs	13419(20.4)	81.4	3075(20.1)	16494(20.3)	263427(24.7)	86.6	40782(26.9)	304209(24.9)
35–44 yrs	20576(31.3)	80.7	4913(32.1)	25489(31.4)	213337(20)	88.1	28904(19)	242241(19.9)
45–54 yrs	16463(25)	78.4	4530(29.6)	20993(25.9)	156723(14.7)	88.8	19784(13)	176507(14.5)
>54 yrs	9515(14.5)	83.6	1869(12.2)	11384(14)	217263(20.3)	90.3	23350(15.4)	240613(19.7)
Median[IQR]	41.8 [33.3–50]		42.6 [34.6–50]	42 [33.6–50]	37.2 [26.9–51.4]		33.9 [24.8–47.2]	36.8 [26.5–50.9]
**Age at 1st +ve /last -ve test** ^a^								
<25 yrs	4522(6.7)	82	991(6)	5513(6.6)	167084(15.6)	81.9	36872(24.3)	203956(16.7)
25–34 yrs	13996(20.7)	80.5	3382(20.6)	17378(20.7)	261316(24.5)	86.4	41202(27.1)	302518(24.8)
35–44 yrs	21714(32.1)	80.3	5337(32.5)	27051(32.2)	224905(21.1)	88.4	29412(19.4)	254317(20.8)
45–54 yrs	17340(25.6)	78.7	4699(28.6)	22039(26.2)	171532(16.1)	89.4	20393(13.4)	191925(15.7)
>54 yrs	10153(15)	83.6	1997(12.2)	12150(14.4)	243355(22.8)	91.1	23892(15.7)	267247(21.9)
Median[IQR]	42.3 [34.2–50.2]		42.3 [34.4–49.8]	42.3 [34.2–50.2]	39.4 [28.9–53.4]		34.3 [25.2–47.7]	38.7 [28.4–52.8]
**Gender**								
Female	23558(34.8)	80.6	5680(31.6)	29238(34.1)	586985(54.9)	89.9	66207(41.8)	653192(53.3)
Male	44162(65.2)	79.4	11485(63.9)	55647(64.9)	481074(45)	85.1	84115(53.1)	565189(46.1)
Other	0(0)	0	6(0)	6(0)	0(0)	0	63(0)	63(0)
Unknown	6(0)	0.7	800(4.5)	806(0.9)	162(0)	2	7940(5)	8102(0.7)
**Health region (1st +/Last–test** ^a^^**)**^								
Interior	10565(15.6)	91.4	999(5.6)	11564(13.5)	138378(13)	89.6	16100(10.2)	154478(12.6)
Fraser	20323(30)	84.6	3705(20.6)	24028(28)	378186(35.4)	92.2	31937(20.2)	410123(33.4)
Vancouver Coastal	18154(26.8)	74.2	6306(35.1)	24460(28.5)	324261(30.4)	83.9	62125(39.2)	386386(31.5)
Vancouver Island	13601(20.1)	92.3	1134(6.3)	14735(17.2)	160836(15.1)	90	17809(11.2)	178645(14.6)
Northern	5067(7.5)	91.6	467(2.6)	5534(6.5)	66427(6.2)	88.3	8806(5.6)	75233(6.1)
Unknown	16(0)	0.3	5360(29.8)	5376(6.3)	133(0)	0.6	21548(13.6)	21681(1.8)
**HIV ever**								
Yes	4639(6.8)	91.7	421(2.3)	5060(5.9)	5414(0.5)	92	472(0.3)	5886(0.5)
No	63087(93.2)	78.2	17550(97.7)	80637(94.1)	1062807(99.5)	87.1	157853(99.7)	1220660(99.5)
**HBV ever**								
Yes	2718(4)	93.1	202(1.1)	2920(3.4)	21149(2)	97.9	458(0.3)	21607(1.8)
No	65008(96)	78.5	17769(98.9)	82777(96.6)	1047072(98)	86.9	157867(99.7)	1204939(98.2)
**Active TB ever**								
Yes	467(0.7)	99.6	2(0)	469(0.5)	2270(0.2)	99.5	12(0)	2282(0.2)
No	67259(99.3)	78.9	17969(100)	85228(99.5)	1065951(99.8)	87.1	158313(100)	1224264(99.8)

^**a**^ Individuals with at least one valid lab record. All lab related calculations were based on these individuals only.

In HCV positives, year of birth was unknown for 8.7% of unlinked vs. <1% of those linked, while in HCV negatives the proportions were 4% and <1%. The linkage rate among those with unknown birth year was 1% in positives and 0.3% in negatives. For HCV positives, unlinked individuals were slightly older at the time of their first HCV test (median age: 42.6 vs. 41.8 years) while HCV negative unlinked individuals were younger than linked individuals (median age 33.9 vs. 37.2 years). Age at first HCV positive test was similar between linked and unlinked. Among HCV negatives, the linkage rate increased with age, while among HCV positives, the linkage rate was highest among the youngest (<25 years) and the oldest groups (≥55 years) and lowest in 45–54 years (78.7%). The proportions of those linked vs. unlinked with unknown gender were higher for both positives (4.5% vs 0%) and negatives (5% vs. 0%). In HCV negatives, the proportion of males in unlinked was higher than in linked (53% vs. 45%) while in HCV positives it was slightly lower in unlinked (63.9% vs 65.2%). Those with unknown health region of residence at the first positive test constituted a larger proportion of unlinked compared to linked (30% vs. 0%), with a similar trend in HCV negatives (13.6% vs. 0%). The largest proportion of unlinked HCV cases were from the Vancouver Coastal health region (VCH) (35%) while in the linked group the highest proportion of cases was from Fraser health region (30%). Similar trends were observed in HCV negatives.

Linkage rates for those HCV positive and co-infected with HIV (92%), HBV (93%) and TB (99.6%) were higher than for those not co-infected. Similarly, for HCV negatives, linkage rates were higher for those with any other infection than those without another infection (**Table 3**).

#### HIV Testers

Most of the unlinked HIV positive (92.3%) and negative (85.7%) individuals had their first test before 2005 and a similar proportion (91.6% of positives and 84.8% of negatives) had their last HIV test before 2005. In linked individuals, the majority of positives had their first test before 2005 (76.4%) while the majority of negatives had their first test after 2005 (58.5%). A similar trend was seen for the year of last test (**Table 4**).

**Table 4:** Comparison of linked and unlinked HIV testers.

	HIV Positive				HIV Negative			
	Linked		Unlinked	Overall	Linked		Unlinked	Overall
	N(%)	% linked	N(%)	N(%)	N(%)	% linked	N(%)	N(%)
N	11025	42.1	15152	26177	988817	47.8	1095068	2083803
**Year of 1st HIV test** ^a^								
1992–1995	1553(19.1)	33.2	3118(44.1)	4671(30.8)	56167(5.7)	14.9	320241(32.5)	376408(19.1)
1996–1999	2224(27.4)	51.2	2122(30)	4346(28.6)	129666(13.2)	31.2	285632(29)	415298(21.1)
2000–2005	2431(29.9)	65.3	1289(18.2)	3720(24.5)	220484(22.5)	48	239096(24.2)	459580(23.4)
2006–2013	1914(23.6)	78.2	535(7.6)	2449(16.1)	573747(58.5)	80.3	141102(14.3)	714849(36.4)
**Year of HIV 1**^**st**^ **+ve/ last -ve test** ^a^								
1992–1995	1467(13.8)	25.6	4269(46.1)	5736(28.8)	13079(1.3)	4.2	300192(30.3)	313271(15.8)
1996–1999	2729(25.7)	51	2622(28.3)	5351(26.9)	53893(5.5)	16.2	277986(28.1)	331879(16.8)
2000–2005	3322(31.2)	66	1709(18.4)	5031(25.3)	135782(13.8)	34.2	260915(26.4)	396697(20.1)
2006–2013	3121(29.3)	82.4	666(7.2)	3787(19)	784278(79.5)	83.9	150717(15.2)	934995(47.3)
**Birth year**								
Before 1945	580(5.3)	27.5	1529(10.1)	2109(8.1)	59061(6)	44	75056(6.9)	134117(6.4)
1945–1964	5693(51.6)	37.4	9520(62.8)	15213(58.1)	235406(23.8)	38.2	380760(34.8)	616166(29.6)
1965–1974	3120(28.3)	53.1	2757(18.2)	5877(22.5)	223813(22.6)	39.3	345808(31.6)	569621(27.3)
After 1975	1632(14.8)	68.7	744(4.9)	2376(9.1)	470537(47.6)	65.7	245184(22.4)	715721(34.3)
Unknown	0	0	602(4)	602(2.3)	0	0	48260(4.4)	48260(2.3)
**Age at 1st HIV test** ^a^								
<25 yrs	1439(16.8)	58.6	1017(11.4)	2456(14)	277085(28)	49.2	285559(27.3)	562644(27.6)
25–34 yrs	2671(31.1)	42.2	3660(41.1)	6331(36.2)	333518(33.7)	45.8	395288(37.8)	728806(35.8)
35–44 yrs	2664(31)	49.1	2766(31.1)	5430(31.1)	177835(18)	45.6	212001(20.3)	389836(19.2)
45–54 yrs	1241(14.5)	53.4	1084(12.2)	2325(13.3)	97249(9.8)	52.1	89262(8.5)	186511(9.2)
>54 yrs	567(6.6)	60.3	374(4.2)	941(5.4)	103130(10.4)	61.4	64698(6.2)	167828(8.2)
Median[IQR]	35.6 [28–43.5]		34.3 [28.7–41.4]	34.9 [28.5–42.4]	31.1 [24.2–41.5]		30.7 [24.4–38.9]	30.8 [24.3–40]
**Age at +ve/ last -ve test** ^a^								
<25 yrs	975(8.8)	40.7	1422(9.8)	2397(9.4)	164202(16.6)	37.8	270112(25.8)	434314(21.3)
25–34 yrs	3393(30.8)	37	5784(39.9)	9177(36)	342495(34.6)	46.2	398053(38)	740548(36.4)
35–44 yrs	3838(34.8)	44.5	4785(33)	8623(33.8)	232901(23.6)	51.4	220512(21.1)	453413(22.3)
45–54 yrs	1994(18.1)	51.7	1866(12.9)	3860(15.1)	125642(12.7)	57.8	91811(8.8)	217453(10.7)
>54 yrs	824(7.5)	56.6	633(4.4)	1457(5.7)	123572(12.5)	65.1	66165(6.3)	189737(9.3)
Median[IQR]	37.6 [31–45.2]		35.1 [29.4–41.9]	36.2 [30–43.4]	34.6 [27.7–45.1]		31.1 [24.8–39.2]	32.7 [26.1–41.9]
**Gender**								
Female	2308(20.9)	57.5	1703(11.2)	4011(15.3)	597939(60.5)	50.9	576972(52.7)	1174911(56.4)
Male	8716(79.1)	40.5	12790(84.4)	21506(82.2)	390846(39.5)	46.7	446061(40.7)	836907(40.2)
Other	0(0)	0	6(0)	6(0)	0(0)	0	110(0)	110(0)
Unknown	1(0)	0.2	653(4.3)	654(2.5)	32(0)	0	71925(6.6)	71957(3.5)
**Health region(1st +ve/Last -ve)** ^a^								
Interior	654(5.9)	88.9	82(0.5)	736(2.8)	130001(13.1)	49.8	131037(12)	261038(12.5)
Fraser	2237(20.3)	91.5	209(1.4)	2446(9.3)	329424(33.3)	57.4	244977(22.4)	574401(27.6)
Vancouver Coastal	5545(50.3)	72.7	2086(13.8)	7631(29.2)	319996(32.4)	41.9	444095(40.6)	764091(36.7)
Vancouver Island	1134(10.3)	86.2	181(1.2)	1315(5)	144143(14.6)	46.5	166081(15.2)	310224(14.9)
Northern	452(4.1)	84.6	82(0.5)	534(2)	65065(6.6)	48.6	68680(6.3)	133745(6.4)
Unknown	1003(9.1)	7.4	12512(82.6)	13515(51.6)	188(0)	0.5	40198(3.7)	40386(1.9)
**HCV ever**								
Yes	4639(42.1)	91.7	421(2.8)	5060(19.3)	45619(4.6)	90.6	4752(0.4)	50371(2.4)
No	6386(57.9)	30.2	14731(97.2)	21117(80.7)	943198(95.4)	46.4	1090316(99.6)	2033514(97.6)
**HBV ever**								
Yes	825(7.5)	97.2	24(0.2)	849(3.2)	13660(1.4)	96.3	531(0)	14191(0.7)
No	10200(92.5)	40.3	15128(99.8)	25328(96.8)	975157(98.6)	47.1	1094537(100)	2069694(99.3)
**Active TB ever**								
Yes	321(7.5)	97.2	7(0.2)	328(3.2)	3475(0.4)	98.6	51(0)	3526(0.2)
No	10704(92.5)	40.3	15145(99.8)	25849(96.8)	985342(99.6)	47.4	1095017(100)	2080359(99.8)

^**a**^ Individuals with at least one valid lab record.

The linkage rate was highest among those born after 1975 for both HIV positives (67%) and negatives (66%). Most of the unlinked HIV positives (81%) were born in 1945–1974 and a similar proportion (85.2%) of linked HIV positives were also born during this time. In unlinked HIV negative testers, ~73% were born 1945–1974 compared to 52.3% among linked HIV negatives. In HIV positives, at the time of the first HIV test, unlinked individuals were slightly younger than linked individuals (median age 34.6 vs. 35.6 years) while for HIV negatives, the age distribution was similar between linked and unlinked individuals (31.1 vs. 30.7 years). Age at the first HIV positive and age at the last HIV negative test among HIV negatives was higher for linked compared to unlinked individuals (HIV positives: 37.6 vs. 35.1;HIV negatives: 34.6 vs. 31.1 years). The majority of HIV positives in the linked (79%) and unlinked groups (84.4%) were male with an additional 4.3% with unknown gender in the unlinked group. In HIV negatives, there were more females (60.4% vs. 52.7%) and fewer individuals with unknown gender (<0.1% vs. 6.6%) in the linked compared to unlinked groups. In HIV positives, health region was not known for 8.6% of the linked and 80.8% of the unlinked groups. However, in the linked group, residence location for further analyses is available from the Client Roster. Among both HIV positives and negatives who also had another infection, the linkage rate was very high (**Table 4**).

Since HIV linkage rates were higher after 2005 related to reportability and better recording of PHNs, we presented characteristics of linked and unlinked individuals who were tested after 2005 in **S1 Table**. As expected, younger birth cohorts (born 1965–74 and ≥1975) represented most of the positives and negatives. However, their linkage rates were lower compared to older birth cohorts tested during this time frame. In HIV positives, the age group 25–34 years had lower linkage rate at first test and HIV diagnosis compared to other age groups (69% vs >80%). Gender distribution was similar to the entire cohort. Linkage rate by region was >90% with known health region in positives and improved considerably in negatives to >80% for all regions except the VCH where the linkage rate was 72%.

## Discussion

The BC-HTC is likely one of the most comprehensive datasets of HCV and HIV cases and testers linked with medical visits, prescription drugs, hospitalizations, cancers and deaths. The data will facilitate an array of population level analyses related to burden of disease, natural history of infection, co-infections and disease syndemics, health care utilization, costs of illness, and impact of HCV treatment on illness costs, morbidity and mortality. It will allow distinguishing the effects of risk activities and other confounders from the effects of HCV infection to inform policy and programs related to HCV and other diseases included in the dataset.

This paper summarises linkage rates over time and by characteristics of linked and unlinked individuals which will assist in interpretation of future analyses. We found that linkage rates increased over time and were much higher for HCV than HIV testers and for those testing positive, older individuals, females and those not from Vancouver.

The linkage rate for HIV test and case data was low and the linkage yield was much lower in earlier (before 2005) than in recent years (after 2005). During earlier years, many tests were submitted for testing without a PHN or non- nominally and it was not possible to link these data even using probabilistic linkage due to missing identifiers. Linkage rates improved when HIV was made reportable in BC in 2003 and when PHN was recorded for patients who tested nominally on a routine basis since 2006 [18]. The linkage rate for HCV was much higher than for HIV (86% vs. 48%) and also increased over time with a decline in 2004/2005. The decline in the linkage rate in 2000–2005 for both HCV and HIV positives relates to HCV RNA testing done for research studies which did not include identifiers needed for linkage, as evidenced by the majority of RNA tests (65%) occurring among unlinked individuals during 2000–2005 (**Table 3**). This is in agreement with a smaller drop in the linkage rate when using anti-HCV tests or HCV case definition alone, rather than the combination of anti-HCV and RNA (**S2 Fig**).

Other characteristics that were related to lower linkage rates for both HCV and HIV were younger age, male sex and residence in Vancouver. For HCV, this may reflect that the population in Vancouver’s Downtown Eastside (DTES), which includes a large proportion of people who inject drugs (PWID) and are homeless, may not have been captured in the linked dataset due to missing identifiers. For HIV, this may reflect men who have sex with men (MSM) living in Vancouver, who may opt for non-nominal testing due to HIV related stigma [19, 20]. Various studies have reported that MSM and youth are more likely to opt for non-nominal HIV testing where available [21–24]. People with high risk behaviors are typically tested repeatedly over time and an individual may have both nominal and non-nominal tests in the dataset. As the identifying information used for linkage became more complete in recent years, testing records for some of these individuals who were not linked may have been included in the linked group, but we are unable to verify that. Of note, we identified additional HIV cases who were not captured through laboratory/surveillance databases (30% of all cases) by using medical visit and hospitalization data (**S2 Fig**). Other studies have also reported lower linkage rates among socially disadvantaged groups due to incomplete records [25, 26]. A study in England and Wales identified poor reporting of HCV data, especially from people at higher HIV risk presenting to genitourinary medicine clinics in London [26]. In this study, 68% of HCV cases were matched with HIV cases reported to the surveillance system between 1996 and 2003. Similar to our datasets, HCV cases which were matched increased from 50% in 1996 to 78% in 2003 due to an increasing completion rate of identifying information.

Other studies linking various datasets, especially those for HCV and HIV have reported similar or lower linkage rates. In Scotland, 10% of HCV cases between 1995–2005 could not be linked to mortality records due to missing identifiers [27]. In a recent linkage of HCV diagnosis with a clinical database to assess engagement with care, the linkage rate based on identifiers was 89%, similar to our findings [28]. The linkage rate in a study between a blood recipient file and the Nova Scotia Health Card registration file using a probabilistic linkage was 65% [29]. In another study in New Zealand, 11% records of deceased prisoners could not be linked with the national death registry while in Australia the linkage rate for HIV deaths with the national death index with optimal sensitivity and specificity was 82% [30, 31]. The linkage rate of various administrative healthcare datasets included in the BC Linked Heath Datasets (now called Population Data BC) not including laboratory and surveillance data was >95% [32]. In the BC-HTC, the linkage rate with the MoH Client Roster was >99% (**Table 2**). Linkage studies from other subject areas have also reported linkage rates in the range of 50–60%, while others have reported very high rates of 80–90% [33–36]. Put in perspective, our linkage rate using a highly specific criterion of the PHN aided by date of birth and names yielded a linkage rate of more than 80% for HCV which was higher in recent years (90%), although linkage rates for HIV were low.

Non-linkage of records has implications for results of future analyses, with the highest likely impact depending on whether individuals with certain characteristics are more or less likely to be linked and if these characteristics are associated with a higher risk of HCV or HIV acquisition or outcomes. Our results show that in the unlinked HCV population, younger age (of whom most tested negative), males and those from VCH were over represented. This suggests that males with high risk behaviours, likely to be PWID from the DTES of Vancouver, were not linked at the same rate as others and likely will be under represented in the linked cohort, if they were represented, some of their laboratory tests were not captured. As the population in the DTES is marginalized and the rate of HCV infection is high, their non-inclusion may lead to an underestimation of disease burden. This could also impact assessment of disparities in testing, treatment uptake, treatment completion and response rates. As noted above, people with high risk behaviors are tested repeatedly and as a result some records of unlinked individuals may have been included in the linked dataset. This will counterbalance the exclusion and reduce any underestimation of burden; however, we are unable to quantify the magnitude of inclusion of the same individuals in both the unlinked and linked groups. Geographic analysis of the linked dataset with overlaying socioeconomic and marginalization indices will provide further insight into differences in testing and infection rates.

Similar to HCV, the unlinked HIV (both positive and negative) population included residents from Vancouver and male or unknown gender. Since large numbers of HIV cases from earlier years could not be linked due to missing identifiers, the overall burden of HIV and co-infection rates will be underestimated in earlier years. However, recovery of additional cases identified from medical visits and hospitalization databases may overcome the lower linkage rate. Non-linkage still affects HIV negative testers for which we did not have additional data sources for recovery, thus under-representing testing in marginalized populations.

### Limitations and gaps

Currently, we are not able to identify immigrants and Aboriginal peoples in the cohort. During the past few decades there has been increasing immigration from HBV and HCV endemic countries to BC and Canada, and the prevalence of HBV and HCV is likely higher in these groups compared to the rest of the Canadian population. In future, linkages with immigration records and other techniques for characterizing ethnicity, such as name recognition, would improve data on immigrants. Similarly linkage with databases of Aboriginal peoples, such as the First Nations client registry would improve characterization of HBV and HCV disease burden in this population.

In summary, the BC HTC links together multiple datasets on a large number of people in BC to enable assessment HCV, HIV and other co-infections and outcome trends; HCV treatment uptake, completion, and effectiveness; cost of illness and effects of treatment on the cost of illness; and disparities in disease risk, outcomes and treatments to inform policy and programming.

## Supporting Information

S1 Fig Detailed data linkage process, BC HTC. (DOCX)

S2 Fig Distribution of HIV positive subjects included BC-HTC in based on various sources. (DOCX)

S3 Fig HCV laboratory data linkage rate based on anti- HCV tests by year 1992–2013. (DOCX)

S1 Table Comparison of linked and unlinked HIV testers 2005–2013, BC HTC. (DOCX)
