# Supplementary material for: Assessing Hepatitis C Burden and Treatment Effectiveness through the British Columbia Hepatitis Testers Cohort (BC-HTC): Design and Characteristics of Linked and Unlinked Participants
Source: PLoS One. 2016 Mar 8;11(3):e0150176. doi: 10.1371/journal.pone.0150176 (PMC4783072; doi:10.1371/journal.pone.0150176)
Supplement: S1 Fig — (DOCX) [file pone.0150176.s001.docx]

**S1 Fig. Process of data linkage between surveillance/laboratory and administrative datasets, BC-HTC**
