# Supplementary material for: Assessing Hepatitis C Burden and Treatment Effectiveness through the British Columbia Hepatitis Testers Cohort (BC-HTC): Design and Characteristics of Linked and Unlinked Participants
Source: PLoS One. 2016 Mar 8;11(3):e0150176. doi: 10.1371/journal.pone.0150176 (PMC4783072; doi:10.1371/journal.pone.0150176)
Supplement: S2 Fig — (DOCX) [file pone.0150176.s002.docx]

**S2 Fig. Distribution of HIV positive subjects included in the BC-HTC based on various sources**

**N=11,025
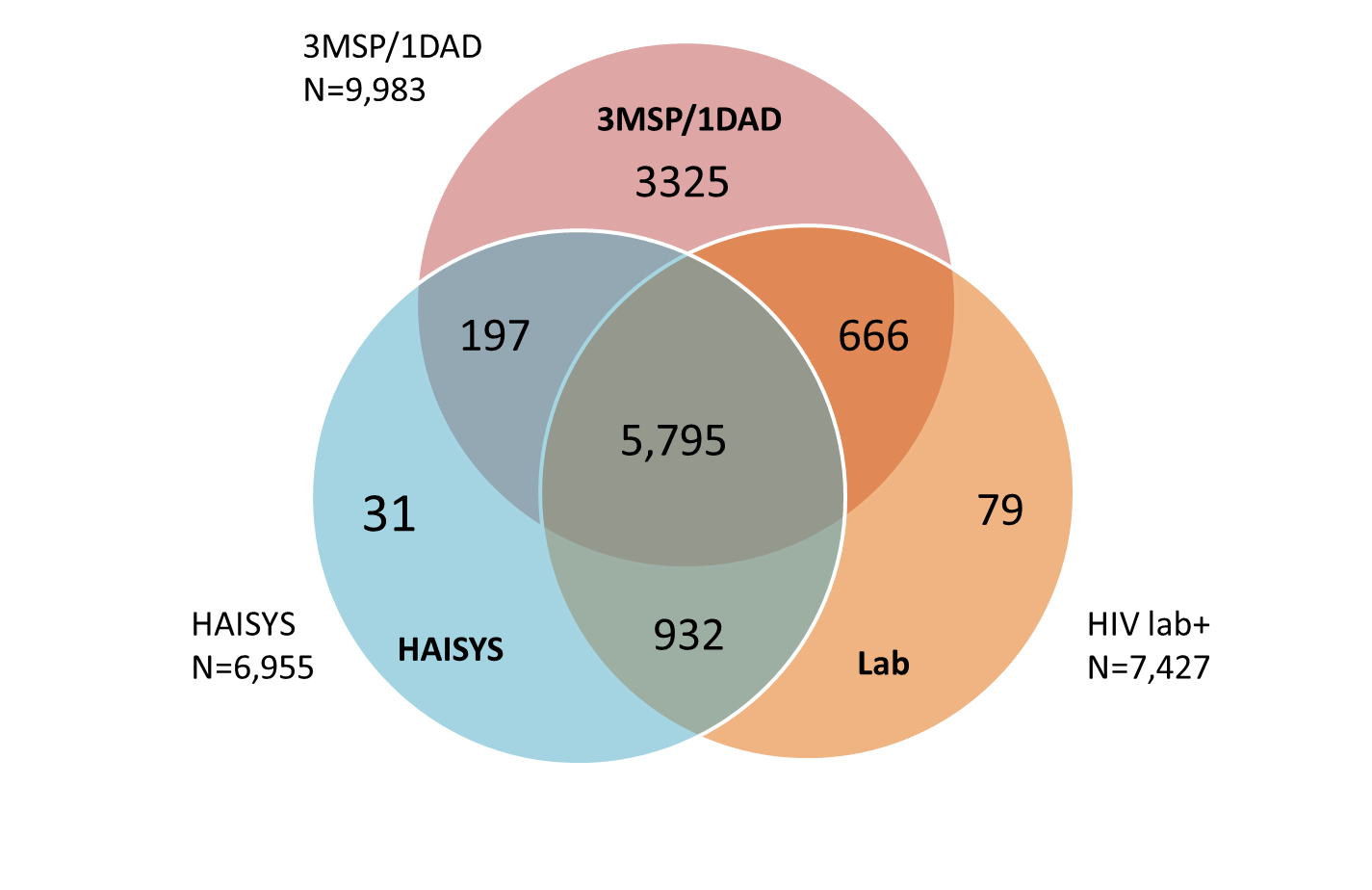
**

MSP: Medical Services Plan; DAD: Discharge Abstract Database; HAISYS: HIV/AIDS Information System
