# Supplementary material for: Assessing Hepatitis C Burden and Treatment Effectiveness through the British Columbia Hepatitis Testers Cohort (BC-HTC): Design and Characteristics of Linked and Unlinked Participants
Source: PLoS One. 2016 Mar 8;11(3):e0150176. doi: 10.1371/journal.pone.0150176 (PMC4783072; doi:10.1371/journal.pone.0150176)
Supplement: S3 Fig — (DOCX) [file pone.0150176.s003.docx]

**S3 Fig. HCV laboratory data linkage rate based on anti- HCV tests by year 1992 - 2013**

Note: Dip in 2004 positives related to specimens from a study without personal identifiers
