# Supplementary material for: Assessing Hepatitis C Burden and Treatment Effectiveness through the British Columbia Hepatitis Testers Cohort (BC-HTC): Design and Characteristics of Linked and Unlinked Participants
Source: PLoS One. 2016 Mar 8;11(3):e0150176. doi: 10.1371/journal.pone.0150176 (PMC4783072; doi:10.1371/journal.pone.0150176)
Supplement: S1 Table — (DOCX) [file pone.0150176.s004.docx]

**S1 Table.** **Comparison of linked and unlinked HIV testers 2005 - 2013, BC-HTC**

|  | **HIV Positive** | | | | **HIV Negatives** | | | |
| --- | --- | --- | --- | --- | --- | --- | --- | --- |
|  | **Linked** | | **Unlinked** | **Overall** | **Linked** | | **Unlinked** | **Overall** |
|  | N(%) | % linked | N(%) | N(%) | N(%) | % linked | N(%) | N(%) |
| N | 2234 | 77.5 | 649 | 2883 | 607901 | 78 | 171684 | 779585 |
| **Birth year** |  |  |  |  |  |  |  |  |
| Before 1945 | 78(3.5) | 83.9 | 15(2.3) | 93(3.2) | 34302(5.6) | 83.8 | 6631(3.9) | 40933(5.3) |
| 1945-1964 | 804(36) | 83.8 | 155(23.9) | 959(33.3) | 107682(17.7) | 79.8 | 27282(15.9) | 134964(17.3) |
| 1965-1974 | 668(29.9) | 79.6 | 171(26.3) | 839(29.1) | 104811(17.2) | 78.6 | 28532(16.6) | 133343(17.1) |
| After 1975 | 684(30.6) | 72.4 | 261(40.2) | 945(32.8) | 361106(59.4) | 78.4 | 99297(57.8) | 460403(59.1) |
| Unknown | 0 | 0 | 47(7.2) | 47(1.6) | 0 | 0 | 9942(5.8) | 9942(1.3) |
| **Age at 1st HIV test** |  |  |  |  |  |  |  |  |
| <25 yrs | 290(13) | 80.1 | 72(12) | 362(12.8) | 161457(26.6) | 76.7 | 49094(30.4) | 210551(27.4) |
| 25-34 yrs | 493(22.1) | 69 | 222(36.9) | 715(25.2) | 208591(34.3) | 79.3 | 54568(33.7) | 263159(34.2) |
| 35-44 yrs | 650(29.1) | 80.9 | 153(25.5) | 803(28.3) | 98574(16.2) | 77.9 | 27978(17.3) | 126552(16.4) |
| 45-54 yrs | 510(22.8) | 84.2 | 96(16) | 606(21.4) | 59948(9.9) | 79.5 | 15438(9.5) | 75386(9.8) |
| >54 yrs | 291(13) | 83.4 | 58(9.7) | 349(12.3) | 79331(13) | 84.4 | 14664(9.1) | 93995(12.2) |
| **Median**  **[range]** | 40.5 [31-49.1] |  | 35.4 [28.4-45.3] | 39.6 [30.2-48.3] | 31.3 [24.6-43.2] |  | 29.9 [23.8-40.7] | 31 [24.4-42.5] |
| **Age at +ve/ last -ve test** |  |  |  |  |  |  |  |  |
| <25 yrs | 271(12.1) | 75.1 | 90(15) | 361(12.7) | 129597(21.3) | 73.3 | 47289(29.2) | 176886(23) |
| 25-34 yrs | 655(29.3) | 74.3 | 226(37.6) | 881(31.1) | 219396(36.1) | 79.8 | 55434(34.3) | 274830(35.7) |
| 35-44 yrs | 651(29.1) | 81.5 | 148(24.6) | 799(28.2) | 113765(18.7) | 80 | 28461(17.6) | 142226(18.5) |
| 45-54 yrs | 420(18.8) | 83.2 | 85(14.1) | 505(17.8) | 62442(10.3) | 79.9 | 15682(9.7) | 78124(10.2) |
| >54 yrs | 237(10.6) | 82 | 52(8.7) | 289(10.2) | 82696(13.6) | 84.8 | 14857(9.2) | 97553(12.7) |
| **Median[IQR]** | 37.8 [29.3-46.8] |  | 34.1 [27.6-43.6] | 37.1 [29-46.3] | 32.7 [26-44.1] |  | 30.1 [24.1-40.9] | 32.2 [25.5-43.3] |
| **Gender** |  |  |  |  |  |  |  |  |
| Female | 432(19.3) | 82.1 | 94(14.5) | 526(18.2) | 372394(61.3) | 83.6 | 73199(42.6) | 432(19.3) |
| Male | 1801(80.6) | 78.1 | 505(77.8) | 2306(80) | 235487(38.7) | 74.2 | 81849(47.7) | 1801(80.6) |
| Other | 0 | 0 | 1(0.2) | 1(0) | 0(0) | 0 | 110(0.1) | 0(0) |
| Unknown | 1(0) | 0.2 | 653(4.3) | 654(2.5) | 32(0) | 0 | 71925(6.6) | 71957(3.5) |
| **Health region(1st +ve/Last -ve)** |  |  |  |  |  |  |  |  |
| Interior | 138(6.2) | 97.2 | 4(0.6) | 142(4.9) | 81954(13.5) | 80.8 | 19459(11.3) | 101413(13) |
| Fraser | 439(19.7) | 100 | 0 | 439(15.2) | 200225(32.9) | 85.1 | 35088(20.4) | 235313(30.2) |
| Vancouver Coastal | 873(39.1) | 93.1 | 65(10) | 938(32.5) | 206092(33.9) | 72.1 | 79732(46.4) | 285824(36.7) |
| Vancouver Island | 192(8.6) | 97 | 6(0.9) | 198(6.9) | 79272(13) | 81 | 18647(10.9) | 97919(12.6) |
| Northern | 108(4.8) | 94.7 | 6(0.9) | 114(4) | 40247(6.6) | 82 | 8842(5.2) | 49089(6.3) |
| Unknown | 484(21.7) | 46 | 568(87.5) | 1052(36.5) | 111(0) | 1.1 | 9916(5.8) | 10027(1.3) |
| **HCV ever** |  |  |  |  |  |  |  |  |
| Yes | 488(21.8) | 94.6 | 28(4.3) | 516(17.9) | 13010(2.1) | 93.2 | 955(0.6) | 13965(1.8) |
| No | 1746(78.2) | 73.8 | 621(95.7) | 2367(82.1) | 594891(97.9) | 77.7 | 170729(99.4) | 765620(98.2) |
| **HBV ever** |  |  |  |  |  |  |  |  |
| Yes | 101(4.5) | 98.1 | 2(0.3) | 103(3.6) | 6466(1.1) | 98 | 129(0.1) | 6595(0.8) |
| No | 2133(95.5) | 76.7 | 647(99.7) | 2780(96.4) | 601435(98.9) | 77.8 | 171555(99.9) | 772990(99.2) |
| **Active TB ever** |  |  |  |  |  |  |  |  |
| Yes | 41(4.5) | 98.1 | 0(0.3) | 41(3.6) | 1702(0.3) | 99.5 | 8(0) | 1710(0.2) |
| No | 2193(95.5) | 76.7 | 649(99.7) | 2842(96.4) | 606199(99.7) | 77.9 | 171676(100) | 777875(99.8) |
